# Reliability and applications of statistical methods based on oligonucleotide frequencies in bacterial and archaeal genomes

**DOI:** 10.1186/1471-2164-9-104

**Published:** 2008-02-28

**Authors:** Jon Bohlin, Eystein Skjerve, David W Ussery

**Affiliations:** 1 Norwegian School of Veterinary Science, P.O. Box 8146 Dep., N-0033 Oslo, Norway; 2 Center for Biological Sequence Analysis, Department of Systems Biology, Technical University of Denmark, DK-2800 Lyngby, Denmark

## Abstract

**Background:**

The increasing number of sequenced prokaryotic genomes contains a wealth of genomic data that needs to be effectively analysed. A set of statistical tools exists for such analysis, but their strengths and weaknesses have not been fully explored. The statistical methods we are concerned with here are mainly used to examine similarities between archaeal and bacterial DNA from different genomes. These methods compare observed genomic frequencies of fixed-sized oligonucleotides with expected values, which can be determined by genomic nucleotide content, smaller oligonucleotide frequencies, or be based on specific statistical distributions. Advantages with these statistical methods include measurements of phylogenetic relationship with relatively small pieces of DNA sampled from almost anywhere within genomes, detection of foreign/conserved DNA, and homology searches. Our aim was to explore the reliability and best suited applications for some popular methods, which include relative oligonucleotide frequencies (ROF), di- to hexanucleotide zero'th order Markov methods (ZOM) and 2.order Markov chain Method (MCM). Tests were performed on distant homology searches with large DNA sequences, detection of foreign/conserved DNA, and plasmid-host similarity comparisons. Additionally, the reliability of the methods was tested by comparing both real and random genomic DNA.

**Results:**

Our findings show that the optimal method is context dependent. ROFs were best suited for distant homology searches, whilst the hexanucleotide ZOM and MCM measures were more reliable measures in terms of phylogeny. The dinucleotide ZOM method produced high correlation values when used to compare real genomes to an artificially constructed random genome with similar %GC, and should therefore be used with care. The tetranucleotide ZOM measure was a good measure to detect horizontally transferred regions, and when used to compare the phylogenetic relationships between plasmids and hosts, significant correlation *(R*^2 ^= *0.4) *was found with genomic GC content and intra-chromosomal homogeneity.

**Conclusion:**

The statistical methods examined are fast, easy to implement, and powerful for a number of different applications involving genomic sequence comparisons. However, none of the measures examined were superior in all tests, and therefore the choice of the statistical method should depend on the task at hand.

## Background

Categorising organisms has always been a key activity in biology. Numerous methods exist, and each reflects particular properties of the organism studied [1]. For microbial organisms, genome sequences are now commonly used as a basis for determining phylogenetic relationships. The simple comparison of nucleotide content (AT/GC content) between species is probably the most used and one of the easiest methods for whole genome comparisons. AT/GC content however, will not necessarily provide reliable information about phylogenetic relationships. It has been proposed that the chromosomal average GC content is more reflective of the environment in which the organism lives, and sometimes less related to its taxonomic group [2,3]. This is in part due to differences in codon usage, which has been presumed to be reflective of the environment in many ways, such as the organism's growth temperature [4], size of the genome and optimal growth rate [5], and anaerobic or aerobic growth conditions [6]. Thus, the amino acid composition and GC content of an organism seems to be related to its ecological niche [7]. Since oligonucleotide usage is influenced by GC content, the habitat of an organism may be largely responsible for the observed DNA word frequencies in genomic DNA.

In most bacteria, the distribution of guanine compared to cytosine on the same strand (GC-skew) varies along the chromosome [8,9], with G's generally biased towards the replication leading strand for nearly all genomes studied so far. Skews in guanine compared to cytosine, or to a lesser extent adenine versus thymine, may indicate where and how DNA replication is taking place and can help identifying the replication leading or lagging strands, as well as the origin and terminus of replication [8,9]. Although this method works for many prokaryotes some have skews that are much less pronounced or even undetectable. Worning and coworkers [8] found that scoring strand bias frequencies of progressively larger oligonucleotides, from dinucleotides up to heptanucleotides, and their reverse complements, the chromosomal location of the origin and terminus of DNA replication could be predicted for nearly all sequenced prokaryotic genomes. Weinel *et al*. [10] have looked at the over- and under-representation of oligonucleotides up to 14-mers to compare two different species of *Pseudomonas*. Abe and colleagues [11] used an algorithm based on neural networks to classify DNA of unknown source by focusing on tetranucleotide frequency distributions. Distributions of oligonucleotides can therefore be used as a more refined way of discriminating between different bacterial genomes.

Samuel Karlin and coworkers [12] applied odds-ratios consisting of observed dinucleotide frequencies divided by expected, determined by genomic nucleotide content, to compare genomes to each other. This method actually dates back to the 1960s [13], but its use has become more popular due to the growing amount of sequenced data available. These relative abundance patterns, or Zero Order Markov distributions (ZOMs), were coined *genomic signatures *by Karlin and coworkers [12]. The reason for calling these distributions "genomic signatures" was that ZOMs were thought to reflect species-specific properties not directly found from GC content or oligonucleotide distributions. Further, ZOMs were found to vary much less within than between even closely related genomes, almost regardless of where DNA was sampled from within the chromosome. ZOMs indicate if oligonucleotides are over- or under-represented in DNA sequences compared with what is expected from average genomic nucleotide content. Differences between observed and expected oligonucleotide distributions might reflect DNA structural conformations, such as DNA curvature, flexibility, and "meltable" regions, in addition to transcription/restriction and other protein binding sites [14-17].

While Karlin and others [12-14] focused on dinucleotide ZOMs, Pride and colleagues [15] compared tetranucleotide ZOMs and tetranucleotide distributions predicted by a Markov Chain Model (MCM) to 16S rRNA based trees to investigate which of the methods had the strongest phylogenetic signal. The Markov chain method assumes that tetranucleotide frequencies have the same likelihood of occurrence, given trinucleotide frequencies, as trinucleotide frequencies have, given dinucleotide frequencies. Alternatively, this can be considered as a tetranucleotide frequency approximation with overlapping trinucleotide frequencies normalized with dinucleotide frequencies. A thorough investigation of how Markov chains approximate oligonucleotides in *Escherichia coli *can be found in [18]. Even though the tetranucleotide MCM is calculated from observed di- and trinucleotide frequencies, the ZOM method, which only uses nucleotide content to approximate tetranucleotides, showed a better congruency towards the 16S rRNA based trees than the MCM method [15]. It was also demonstrated that tetranucleotide ZOMs differed in coding and non-coding areas in contrast to dinucleotide ZOMs [15].

It is the goal of this paper to examine the strengths and weaknesses of a series of DNA sequence comparison methods, based on statistical distributions of oligonucleotides. These methods include Relative Oligonucleotide Frequencies (ROFs), di- to hexanucleotide ZOMs and tetranucleotide MCMs. In the literature the focus has mainly been on di- [12-14] and tetranucleotide ZOMs [15], and tetranucleotide ROFs [11] and MCMs [12,14,15,19]. In this work, tri-, penta- and hexanucleotide ZOMs are included additionally to see how they compare against the more popular measures and whether they have any useful differences. The different measures are compared against each other, both as tools for DNA profiling and sequence comparisons, and the most appropriate measure is used to look at plasmid-host relations as an application.

## Results

### Comparing the phylogenetic strength of the statistical measures using a random DNA sequence

**Figure 1:**
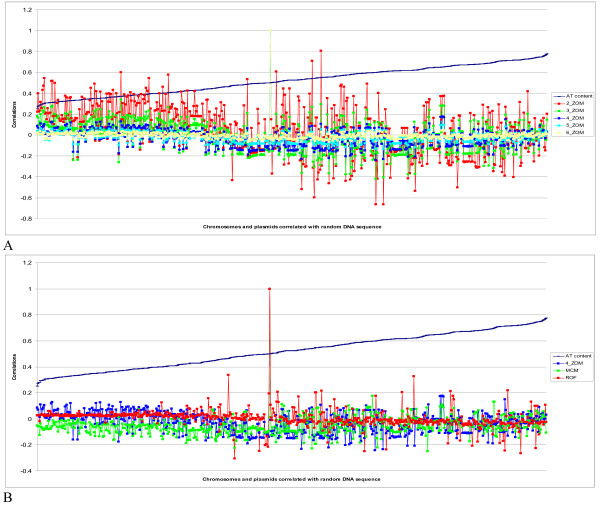
**Random genome compared to sequenced bacterial genomes**. Comparisons between 581 sequenced bacterial and archaeal chromosomes and plasmids with a random 5.3 mbp DNA sequence with 50% GC content. The comparisons were performed to test the reliability of different oligonucleotide based statistical measures consisting of di- to hexanucleotide ZOMs, tetranucleotide ROFs and MCMs. The chromosomes and plasmids, represented as points along the horizontal axis, were correlated with the random DNA sequence, with the corresponding correlation scores on the vertical axis, and sorted by increasing AT content from left to right. Higher correlation scores means better match. In (A) all chromosomes and plasmids were compared using di- to hexanucleotide ZOMs, while in (B) they were compared using tetranucleotide ROFs and MCMs, with tetranucleotide ZOMs included as reference. It can be observed that dinucleotide ZOMs achieve surprisingly high correlation scores (A) while hexanucleotide ZOMs show no correlation at all. Tetranucleotide ROFs (B) achieves slightly higher correlation values than both tetranucleotide MCMs and ZOMs.

In the first test, all the measures mentioned above were used to compare 581 bacterial and archaeal chromosomes and plasmids with a completely random DNA sequence with similar size and GC content to an *E. coli *chromosome, which for this case can be considered as a 5 mbp genome with 50% GC. Lower correlation values between genomes and the random DNA sequences means that the method is more reliable with fewer false positives. For all measures, correlation values above 0.7 indicate some kind of relation. From Figure 1(A) (see additional file 1 for further details) it can be observed that the dinucleotide ZOMs had, by far, the largest spread with respect to correlation scores ranging from -0.66 for *Pseudoalteromonas haloplanktis *strain TAC125 to *Methanospirillum hungatei *strain JF-1 with a correlation of 0.81. Stepping up to trinucleotide ZOMs the spread decreased dramatically with *P. haloplanktis *strain TAC125, again having the lowest correlation value of -0.42, while *Corynebacterium efficiens *strain YS-314 had the highest value of 0.34. The tetranucleotide ZOMs were still better, with scores ranging from -0.24 for *P. haloplanktis *strain TAC125 to 0.18 for *Leptospira interrogans *serovar Copenhageni. Pentanucleotide ZOM correlations ranged from -0.13 to 0.1, while hexanucleotide ZOMs were firmly placed with the lowest correlation scores, never going outside the interval (-0.07, 0.05) for all 581 DNA sequences tested. Turning now to tetranucleotide ROFs and MCMs, as shown in Figure 1(B), the correlation scores were between (-0.31, 0.34), and (-0.25, 0.18), respectively. For the ROF measure mostly plasmids were found at the lower end of the correlation score interval. *Xylella fastidiosa *strain 9a5c and *Geobacter metallireducens *strain GS-15 were the first chromosomes from the lower end of the correlation list, obtaining scores of -0.17 and 0.05, respectively. Tetranucleotide MCMs found *Sulfolobus solfataricus *strain P2 and *Acinetobacter *sp. ADP1 at each end, respectively, in the correlation score interval (-0.25, 0.18).

### Comparing the measures using real genomes

We used *Bacillus subtilis *subsp. subtilis str. 168, *Yersinia pestist *biovar Microtus str. 91001 (former Medievalis), *Pseudomonas aeruginosa *strain PAO1 and *Staphylococcus areus *subsp. *aureus *strain Mu50, to compare the phylogenetic capabilities of the different statistical measures. Additionaly, the measures were used to assess possible phylogenetic relationships of *T. maritima*, which has been difficult to classify due to extensive horizontal transfer [20-22].

Starting with *B. subtilis*, see Table 1 for details concerning strains, correlation scores, GC content and size, the dinucleotide ZOM measure found the *γ*-proteobacteria *Hahella chejuensis *strain KCTC 2396, and firmicutes *Bacillus licheniformis *strain ATCC 14580, *Bacillus cereus *strain ATCC 10987, *Bacillus anthracis *strain 'Ames Ancestor', and *Bacillus thuringiensis *subsp. *konkukian *serotype H34 strain 97-27 ranked after plasmid pHCM1 belonging to *γ*-proteobacterium *Salmonella enterica *subsp. *enterica *serovar Typhi strain CT18. The trinucleotide ZOM measure found *B. licheniformis *as the closest match, followed by a set of genomes and plasmids of proteobacteria.

**Table 1:** *B. subtilis *compared to 581 genomic DNA sequences using ZOM, MCM and ROF measures

**Name**	**Genbank**	**Size mbp**	**AT**	**2-ZOM**	**3-ZOM**	**4-ZOM**	**5-ZOM**	**6-ZOM**	**MCM**	**ROF**
*Bacillus subtilis *subsp. *subtilis *strain 168	AL009126.2	4.21	0.56	1	1	1	1	1	1	1
*Hahella chejuensis *strain KCTC 2396	CP000155.1	7.22	0.46	0.96	0.86	0.80	0.75	0.71	0.51	0.34
*Salmonella enterica *subsp. *enterica *serovar Typhi strain CT18, plasmid pHCM1	AL513383	0.22	0.52	0.96	0.89	0.83	0.78	0.72	0.46	0.85
*Bacillus licheniformis *strain ATCC 14580	AE017333.1	4.22	0.54	0.95	0.96	0.96	0.95	0.94	0.90	0.95
*Bacillus cereus *strain ATCC 10987	AE017194.1	5.22	0.64	0.95	0.74	0.69	0.67	0.64	0.71	0.80
*Bacillus anthracis *strain 'Ames Ancestor'	AE017334.2	5.23	0.65	0.95	0.73	0.69	0.66	0.64	0.71	0.79
*Bacillus thuringiensis *subsp. *konkukian *serotype H34 strain 97-27	AE017355.1	5.24	0.65	0.95	0.74	0.69	0.66	0.64	0.71	0.79
*Bacillus clausii *strain KSM-K16	AP006627.1	4.30	0.55	0.95	0.85	0.82	0.80	0.78	0.82	0.90
*Geobacillus kaustophilus *strain HTA426	BA000043.1	3.54	0.48	0.87	0.82	0.80	0.79	0.77	0.82	0.59

*B. licheniformis *was the closest genome for tetra- to hexanucleotide ZOMs. The penta- and hexanucleotide ZOMs ranked *Bacillus clausii *strain KSM-K16 under *B. licheniformis*. The same was observed for the tetranucleotide MCM measure, but in contrast to the ZOM measures, a set of *Bacillus *species followed, and genera from other phyla obtained only low correlation scores with this measure.

*B. licheniformis *was also found on the top of the list for the tetranucleotide ROF measure, followed by *Bacillus halodurans *strain C-125 and *Geobacillus kaustophilus *strain HTA426. Bacteria from different phyla populated the list further down. Additional file 2 contains complete listings of comparisons between *B.subtilis *and all genomes for the measures discussed.

For *P. aeruginosa*, see Table 2 (additional file 3 for more information), dinucleotide ZOMs found plasmids from *Ralstonia metallidurans *strain CH34, *Xanthomonas campestris *pv. *vesicatoria *strain 85-10 and *Azoarcus *sp. *EbN1 *as best matches. The best matching chromosome with the dinucleotide ZOM measure was the actinobacterium*Corynebacterium jeikeium *strain K411.

**Table 2:** *P. aeruginosa *compared to 581 genomic DNA sequences using ZOM, MCM and ROF measures

**Name**	**Genbank**	**Size mbp**	**AT**	**2-ZOM**	**3-ZOM**	**4-ZOM**	**5-ZOM**	**6-ZOM**	**MCM**	**ROF**
*Pseudomonas aeruginosa *strain PAO1	AE004091	6.26	0.33	1	1	1	1	1	1	1
*Ralstonia metallidurans *strain CH34, plasmid 1	CP000354	0.23	0.40	0.97	0.87	0.82	0.78	0.72	0.61	0.93
*Xanthomonas campestris *pv. *vesicatoria *strain 85-10, plasmid pXCV183	AM039951	0.18	0.40	0.97	0.87	0.84	0.80	0.74	0.76	0.93
Azoarcus sp. EbN1, plasmid 2	CR555308	0.22	0.37	0.96	0.89	0.87	0.84	0.80	0.85	0.95
*Corynebacterium jeikeium *strain K411	CR931997.1	2.46	0.39	0.95	0.91	0.88	0.84	0.81	0.79	0.95
*Pseudomonas entomophila *strain L48	CT573326.1	5.89	0.36	0.89	0.92	0.93	0.93	0.93	0.96	0.96
*Pseudomonas fluorescens *strain Pf-5	CP000076.1	7.07	0.37	0.85	0.88	0.90	0.90	0.90	0.95	0.94
*Pseudomonas fluorescens *strain PfO-1	CP000094.1	6.44	0.39	0.91	0.87	0.86	0.86	0.85	0.87	0.93
*Ralstonia eutropha *strain H16, chromosome 1	AM260479	4.05	0.34	0.92	0.90	0.90	0.90	0.88	0.90	0.97
*Ralstonia solanacearum *strain GMI1000	AL646052.1	3.72	0.33	0.93	0.90	0.90	0.89	0.88	0.92	0.96
*Ralstonia eutropha *strain JMP134	CP000091.1	3.81	0.35	0.92	0.90	0.89	0.88	0.87	0.88	0.95
*Pseudomonas putida*_strain KT2440	CP000712.1	6.18	0.38	0.82	0.85	0.86	0.87	0.86	0.90	0.92
*Novosphingobium aromaticivorans *strain DSM 12444	CP000248.1	3.56	0.35	0.93	0.91	0.88	0.86	0.84	0.80	0.95

The ZOM measures from trinucleotides and up ranked *Pseudomonas entomophila *strain L48 highest while the *α*-proteobacterium *Novosphingobium aromaticivorans *strain DSM 12443 was the third best match.

Although all ZOM measures found the *β*-proteobacteria *Ralstonia sp*. relatively close to *P. aeruginosa*, only penta- and hexanucleotide ZOMs ranked *Pseudomonas fluorescens *strain Pf-5 as the third best match.

*P. aeruginosa *was found closest to *P. entomophila *and *P. fluorescens *with the MCM measure, while the *β*-proteobacterium *Ralstonia solanacearum *strain GMI1000 followed close behind. Other species of *Pseudomonas *obtained relatively high correlation scores, but were more dispersed, with respect to ranking, than the results from the penta- and hexanucleotide ZOMs.

The ROF measure found *Ralstonia eutropha *strain H16 closest to *P. aeruginosa*, and, with the exception of *P. entomophila*, other species of *Pseudomonas *were more dispersed for this measure than for any of the other tested. Soil bacteria in general obtained high correlation scores for all the measures tested.

Other strains of *S. aureus *ranked as closest matches to *S. aureus *subsp. *aureus *strain Mu50, see Table 3 (or additional file 4 for complete listings), with high correlation scores for all measures. *Staphylococcus saprophyticus *subsp. *saprophyticus *strain ATCC 15305 ranks after all *S. aureus *strains for all ZOM measures, while *Staphylococcus haemolyticus *strain JCSC 1435 followed for tri- to hexanucleotide ZOMs. Two strains of *Staphylococcus epidermidis *were only found together with *S. aureus *and *S. haemolyticus *for tetra- to hexanucleotide ZOMs. The *γ*-proteobacterium *Acinetobacter *sp. ADP1 was found closer to *S. aureus *than *S. haemolyticus *and *S.epidermidis *for the dinucleotide ZOM measure. All ZOM measures ranked a set of proteobacteria like *Photobacterium profundum *strain SS9 and *Vibrio fischerii *strain ES114 before other members of the firmicutes phyla.

**Table 3:** *S. aureus *compared to 581 genomic DNA sequences using ZOM, MCM and ROF measures

**Name**	**Genbank**	**Size mbp**	**AT**	**2-ZOM**	**3-ZOM**	**4-ZOM**	**5-ZOM**	**6-ZOM**	**MCM**	**ROF**
*Staphylococcus aureus *subsp. *aureus *strain Mu50	BA000017.4	2.88	0.67	1	1	1	1	1	1	1
*Staphylococcus aureus *subsp. *aureus *strain N315	BA000018.3	2.81	0.67	0.99	0.99	0.99	0.99	0.99	0.99	0.99
*Staphylococcus aureus *subsp. *aureus *strain USA300	CP000255.1	2.87	0.67	0.99	0.99	0.99	0.99	0.99	0.99	0.99
*Staphylococcus aureus *subsp. *aureus *strain MSSA476	BX571857.1	2.80	0.67	0.99	0.99	0.99	0.99	0.99	0.99	0.99
*Staphylococcus aureus *subsp. *aureus *strain MW2	BA000033.2	2.82	0.67	0.99	0.99	0.99	0.99	0.99	0.99	0.99
*Staphylococcus aureus *subsp. *aureus *strain COL	CP000046.1	2.81	0.67	0.99	0.99	0.99	0.99	0.99	0.99	0.99
*Staphylococcus aureus *subsp. *aureus *strain NCTC 8325	CP000253.1	2.82	0.67	0.99	0.99	0.99	0.99	0.99	0.99	0.99
*Staphylococcus aureus *subsp. *aureus *strain MRSA252	BX571856.1	2.90	0.67	0.99	0.99	0.99	0.99	0.99	0.99	0.99
*Staphylococcus aureus *strain RF122	AJ938182.1	2.74	0.67	0.99	0.99	0.99	0.99	0.99	0.99	0.99
*Staphylococcus haemolyticus *strain JSCS 1435	AP006716.1	2.69	0.67	0.98	0.96	0.96	0.95	0.93	0.97	0.99
*Staphylococcus epidermidis *strain RP62A	CP000029.1	2.62	0.68	0.90	0.90	0.92	0.92	0.91	0.96	0.99
*Staphylococcus epidermidis *strain ATCC 12228	AE015929.1	2.50	0.68	0.92	0.91	0.92	0.92	0.91	0.96	0.99
*Staphylococcus saprophyticus *subsp. *saprophyticus *strain ATCC 15305	AP008934.1	2.52	0.67	0.99	0.98	0.98	0.97	0.95	0.95	0.99
*Oceanobacillus iheyensis *strain THE 831	BA000028.3	3.63	0.64	0.58	0.52	0.59	0.61	0.61	0.87	0.96
*Acinetobacter *train ADP1	CR543861	3.60	0.60	0.98	0.92	0.83	0.77	0.73	0.32	0.89
*Photobacterium profundum *strain SS9, chromosome 2	CR354532	2.24	0.59	0.97	0.90	0.84	0.78	0.73	0.58	0.92
*Photobacterium profundum *strain SS9, chromosome 1	CR354531	4.09	0.58	0.96	0.91	0.86	0.80	0.76	0.60	0.89
*Vibrio fischeri *strain ES114, chromosome 2	CP000021	1.33	0.63	0.96	0.90	0.86	0.82	0.77	0.69	0.97
*Vibrio fischeri *strain ES114, chromosome 1	CP000020	2.91	0.61	0.94	0.89	0.86	0.82	0.79	0.69	0.94

*S. aureus *was found close together with other species and genera of firmicutes, just as *B. subtilis *was, with the MCM measure. Other species of *Staphylococcus *ranked highest, followed by *Oceanobacillus iheyensis *strain HTE831.

Members of the firmicutes phylum were also predominantly present as the best matching members when the ROF measure was used, with only the second chromosome of *V. fischerii *breaking this trend. *V. fischerii*, and *Vibrio *sp. in general, obtained high correlation scores for all measures except the MCM.

For *Y. pestis *biovar Microtus str. 91001, see Table 4 (additional file 5 for more information), most measures found other "enterics" as closest matches, but with some variations in the rankings. *Yersinia pseudotuberculosis *strain IP32953 correlated identically with the other *Yersinia *species, and the relative *Photorhabdus luminescens *subsp. *laumondii *strain TTO1 obtained high correlation scores for all measures. The dinucleotide ZOM measure however, found the bacteroidete *Cytophaga hutchinsonii *strain ATCC 33406 as the best match to the *Yersinia *species. Other members of the proteobacteria phyla followed further down the list. The number of "enterics" close to *Yersinia *sp. increased with word size for the ZOM measures. *Alcanivorax borkumensis *strain SK2 ranked highest after *P. luminescens *for tri- and tetranucleotide ZOMs while *E*. *coli *were closer for penta- and hexanucleotide ZOMs. The MCM measure ranked *Erwinia carotovora *subsp. *atroseptica *strain SCRI 1043 as best match to *Yersinia *sp., followed closely by *P. luminescens*. All "enterics" obtained relatively high correlation scores with the MCM measure.

**Table 4:** *Y. pestis *compared to 581 genomic DNA sequences using ZOM, MCM and ROF measures

**Name**	**Genbank**	**Size mbp**	**AT**	**2-ZOM**	**3-ZOM**	**4-ZOM**	**5-ZOM**	**6-ZOM**	**MCM**	**ROF**
*Yersinia pestis *biovar Microtus strain 91001	AE017042	4.60	0.52	1	1	1	1	1	1	1
*Yersinia pestis *strain Nepal516	CP000305.1	4.53	0.52	0.99	0.99	0.99	0.99	0.99	0.99	0.99
*Yersinia pestis *strain KIM	AE009952.1	4.60	0.52	0.99	0.99	0.99	0.99	0.99	0.99	0.99
*Yersinia pestis *strain CO92	AL590842.1	4.65	0.52	0.99	0.99	0.99	0.99	0.99	0.99	0.99
*Yersinia pseudotuberculosis *strain IP32953	BX936398.1	4.74	0.52	0.99	0.99	0.99	0.99	0.99	0.99	0.99
*Yersinia pestis *strain Antiqua	CP000308.1	4.70	0.52	0.99	0.99	0.99	0.99	0.99	0.99	0.99
*Cytophaga hutchinsonii *strain ATCC 33406	CP000383.1	4.43	0.61	0.97	0.78	0.71	0.66	0.63	0.50	0.70
*Photorhabdus luminescens *subsp. *laumondi *strain TTO1	BX470251.1	5.69	0.57	0.97	0.97	0.96	0.94	0.93	0.91	0.87
*Sodalis glossinidius *strain 'morsitans', plasmid pSG1	AP008233	0.08	0.51	0.96	0.92	0.88	0.85	0.79	0.78	0.86
*Alcanivorax borkumensis *strain SK2	AM286690.1	3.12	0.45	0.96	0.92	0.90	0.88	0.86	0.76	0.60
*Escherichia coli *strain K-12	U00096.2	4.64	0.49	0.90	0.90	0.89	0.88	0.87	0.81	0.81

The ROF measure ranked *Sodalis glossinidius *strain 'morsitans' as closest match to *P. luminescens*, and *Yersinia *sp. plasmids ranked higher with this measure than the others.

Comparing the different measures using *T. maritima*, see Table 5 (additional file 6 for complete listings), tetranucleotide ZOMs found the following bacteria closest: *Archaeoglobus fulgidus *strain DSM 4304, *Geobacter metallireducens *strain GS 15, and *Thermococcus kodakaraensis *strain KOD1. A 36 kb plasmid of *Salinibacter ruber *strain DSM 13855, pSR35, ranked after *T. kodakaraensis*. All tested oligonucleotide ZOMs ranked the thermophilic euryarchaeota *T. kodakaraensis *highest.

**Table 5:** *T. maritima *compared to 581 genomic DNA sequences using ZOM, MCM and ROF measures

**Name**	**Genbank**	**Size mbp**	**AT**	**2-ZOM**	**3-ZOM**	**4-ZOM**	**5-ZOM**	**6-ZOM**	**MCM**	**ROF**
*Thermotoga maritima *strain MSB 8	AE000512.1	1.86	0.54	1	1	1	1	1	1	1
*Thermococcus kodakaraensis *strain KOD1	AP006878.1	2.09	0.48	0.88	0.81	0.76	0.73	0.69	0.72	0.62
*Salinibacter ruber *strain DSM 13855	CP000160	0.04	0.42	0.88	0.80	0.75	0.68	0.56	0.38	0.20
*Archaeoglobus fulgidus *strain DSM 4304	AE000782.1	2.18	0.51	0.82	0.76	0.71	0.67	0.63	0.64	0.72
*Salinibacter ruber *strain DSM 13855	CP000159.1	3.55	0.34	0.79	0.70	0.66	0.64	0.62	0.42	-0.11
*Geobacter metallireducens *strain GS 15	CP000148.1	4.0	0.40	0.76	0.77	0.74	0.70	0.67	0.69	0.16
*Aquifex aeolicus *strain VF5	AE000657.1	1.55	0.57	0.66	0.67	0.66	0.65	0.63	0.58	0.70
*Chlamydia trachomatis *strain A/HAR-13	CP000051	1.04	0.59	0.68	0.68	0.67	0.64	0.61	0.43	0.68
*Chlamydia trachomatis *strain D/UW-3/CX	AE001273	1.04	0.59	0.68	0.68	0.67	0.64	0.61	0.43	0.68
*Pelobacter carbinolicus *strain DSM 2380	CP000142.2	3.67	0.45	0.39	0.40	0.42	0.41	0.41	0.74	0.18
*Syntrophus aciditrophicus *strain SB	CP000252.1	3.18	0.49	0.68	0.65	0.64	0.63	0.61	0.79	0.59

It is interesting to note that for *T. maritima *the dinucleotide ZOM measure seemed to be under-correlating much more than for the random genome mentioned above.

Tetranucleotide ROFs had *A. fulgidus*, *Aquifex aeolicus *strain VF5 and *Chlamydia trachomatis *strain A HAR-13 as closest matches. The MCM measure ranked *T. kodakaraensis *strain KOD1, *Pelobacter carbinolicus *strain DSM 2380 and *Syntrophus aciditrophicus *strain SB, both *δ*-proteobacteria, on top. *A. aeolicus*, which is assumed to be one of *T. maritima *closest sequenced relatives [20], was ranked progressively higher for di- to hexanucleotide ZOMs, achieving a 36. place for dinucleotide ZOMs and a 6. place for hexanucleotide ZOMs. As was mentioned above, *A. aeolicus *has the second best correlation score for ROFs while tetranucleotide MCMs rank *A. aeolicus *as the 113. closest match with *T. maritima*.

The Markov measures, including both ZOM and MCM methods, seemed to be more similar to each other than to the ROF measure. Considerably higher correlation scores were obtained, on average, for the dinucleotide ZOM measure, while the hexanucleotide ZOM and tetranucleotide MCM measures produced the lowest. The ROF measure seemed to have the most varied average correlation scores.

### Autocorrelation profiles

**Figure 2:**
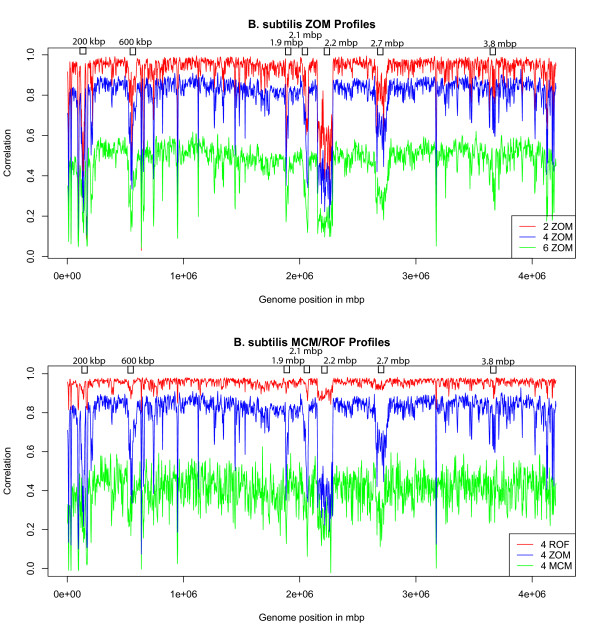
***B. subtilis *tetranucleotide MCM, ROF, and ZOM autocorrelation profiles**. Di-, tetra- and hexanucleotide ZOM (top), respectively red, green and blue lines, together with tetranucleotide MCM and ROF (below), respectively green and red lines, based autocorrelation profiles of *B. subtilis*. Autocorrelation scores (vertical axis) are obtained with 5 kbp sliding windows, overlapping every 2.5 kbp, correlated with mean genomic values. The horizontal axis represents chromosomal position, with each point spanning 5 kbp. Average autocorrelation scores drop progressively for di- to hexanucleotide ZOMs, presumably due to lower departure values between observed and expected tetranucleotide frequencies caused by small sliding windows. ZOM and ROF based profiles appear similar, but the former appear more detailed. Although the hexanucleotide ZOM and tetranucleotide MCM measures had similar average autocorrelation scores, the latter can be observed to vary considerably more than the former. All marked dots represent presumed horizontally acquired DNA, and the two largest dips located close to 2.2 mbp and 2.7 mbp are known prophages.

**Figure 3:**
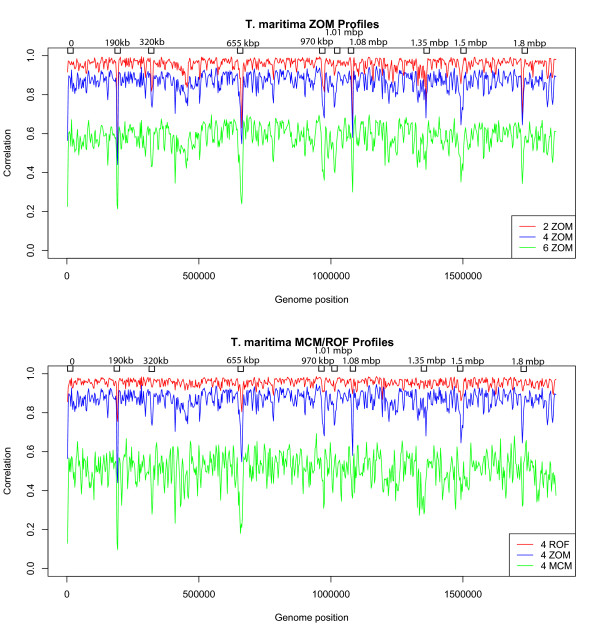
***T. maritima *tetranucleotide MCM, ROF, and ZOM autocorrelation profiles**. Di-, tetra- and hexanucleotide ZOMs (top), respectively red, green and blue lines, together with tetranucleotide MCM and ROF (bottom), respectively green and red lines, based autocorrelation profiles of *T. maritima*. Autocorrelation scores (vertical axis) were obtained with 5 kbp sliding windows, overlapping every 2.5 kbp, correlated with mean genomic values. The horizontal axis represents chromosomal position, with each point spanning 5 kbp. All large dips, except the one found at position 190 kbp, which was found to be 16S, 23S and 5S rRNA genes, are presumed to be horizontally transferred. The marked dips in the tetranucleotide ZOM profiles are part of a presumed horizontally acquired ABC transport system. It can be observed from the Figure that the profile based on tetranucleotide ROFs resembles the ZOM profiles, but that some dips are less visible. The low average autocorrelation value in the tetranucleotide MCM profile is assumed to be caused by lower departure values between observed and expected tetranucleotide frequencies due to small sliding window size. Although many of the large dips found in the other measures were absent in the MCM profile, irregularities (marked dots) were observed in the MCM profile that were not easily detectable with the other measures. Looking at the di-, tetra- and hexanucleotide ZOM profiles, progressively more fluctuations can be observed for increasing oligonucleotide size while average autocorrelation scores drop.

Figures 2 and 3 illustrate some of the above mentioned measures in autocorrelation profiles of *B. subtilis *and *T. maritima*.

The *B. subtilis *genome is presumed to consist of large sections of horizontally acquired DNA [23], and was therefore used to compare the capabilities of the different statistical measures to detect foreign DNA. The autocorrelation profiles in Figure 2 contain a set of dips that may be conserved and/or foreign DNA. BLAST [24] searches against the NCBI *nr *database were used to get information about these special regions, while genes and proteins were examined further with the STRING database [25].

The dips at 200 kbp and 600 kbp, see Figure 2, were presumed to be remnants of prophages, while the remaining visible dips just short of genomic position 1 mbp were conserved proteins and/or rRNA genes according to BLAST. The two dips just before and after position 2 mbp were found to be transcriptional regulators, possibly horizontally transferred [23], while the large dips at positions 2.2 and 2.7 mbp are known prophages [23]. It should be noted that BLAST identified an *uvrX *gene, involved in DNA repair, together with the prophage at position 2.2 mbp. Three more sections were examined more carefully, and they all turned out to contain rRNA genes, except the dip found close to position 3.8 mbp according to BLAST. This dip was fairly easy to observe with the different ZOM measures, but harder with the ROF and MCM measures. A BLAST search resulted in a hit for a *rodC *gene that is active in cell synthesis.

Focusing on the *T. maritima *genome, especially visible (see Figure 3) are the two dips located at positions close to 0 and 1.5 mbp, which are barely detectable from the dinucleotide ZOM profile. Turning to the other measures it can be seen that the tetranucleotide ROF profile has a very high average autocorrelation score, yet the dip found in all autocorrelation ZOM profiles at position close to 1.08 mbp is hardly visible. A similar phenomenon was also observed in the tetranucleotide MCM autocorrelation profile. This profile contained dips that were more pronounced than any of the other profiles, with the ones found close to positions 320 kbp and 1.35 mbp standing particularly out. Selections starting from positions 320 kbp, 970 kbp, 1.08 mbp, and 1.72 mbp all gave hits related to an ABC transporter system that is presumed to have been acquired horizontally from species belonging to the *Archaea *kingdom [20-22]. All the selections were 5 kbp except for the last which was 10 kbp. The selections starting from positions 0, 655 kbp (10 kbp selection), 1.01 mbp, and 1.35 mbp were all termed hypothetical proteins except for the last, which was termed *lacI *family transcriptional regulator (lactose operon repressor [25]), and the first, position 0, which matched *celA *and *celB *genes, which, according to [20,25], encodes glycosyl hydrolases. The biggest dip, found in all autocorrelation profiles above, starts at position 190 kbp and extends 5 kbp, encodes 16S, 23S, and 5S rRNA genes according to BLAST. At position 1.49 mbp another large dip was clearly visible on all autocorrelation profiles, except for dinucleotide ZOM and the MCM measures. BLAST listed a *spc *operon that encodes a set of ribosomial proteins according to [25]. *T. maritima *is believed to have acquired large amounts of DNA from other bacteria and archaea [20-22]. Estimations go as high as 24% from archaea alone and still more DNA is presumed to have been acquired from other prokaryotes, and possibly eukaryotic species as well [20-22]. Homologues to all or parts of the extracted sequences at positions 965 kbp, 1.077 mbp and 1.8 mbp were found in *Pyrococcus furiosus *DSM 3638, *Sinorhizobium meliloti*, and *Clostridium thermocellum *ATCC 27405, respectively.

**Figure 4:**
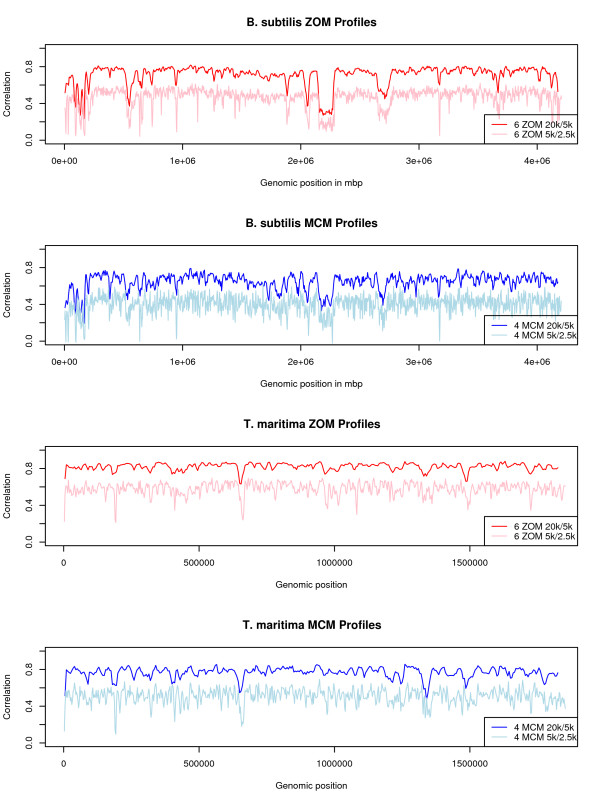
***B. subtilis *and *T. maritima *hexanucleotide ZOM and tetranucleotide MCM autocorrelation profiles**. Hexanucleotide ZOM (red) and tetranucleotide MCM (blue) based autocorrelation profiles. Autocorrelation scores (vertical axis) were obtained with 5 kbp and 20 kbp sliding windows, overlapping every 2.5 kbp and 5 kbp, respectively, and correlated with mean genomic values. The horizontal axis represents genome position and each point of the red and blue curves spans 5 kbp, while each point of the light blue and pink curves spans 20 kbp. It can be observed from the graphs that increasing sliding window size increases average autocorrelation score for both hexanucleotide ZOM and tetranucleotide MCM profiles, but reduces detail. The tetranucleotide MCM measure (blue and light blue curves) had, in general, larger variance for the genomes tested than the hexanucleotide ZOM measure (red and pink curves), implying that the MCM measure was more sensitive to genomic changes.

The hexanucleotide ZOM and tetranucleotide based MCM had the lowest average autocorrelation scores of the measures tested. Increasing the sliding window size from 5 kbp to 20 kbp, overlapping every 5 kbp, did increase average autocorrelation scores, but visual detail was poorer (Figure 4). The ZOM measures were found to be similar, with the tetranucleotide ZOM possibly the best measure of all tested with respect to size and detail.

Other differences include more pronounced dips for tetra- and hexanucleotide ZOMs, and fewer for ROFs, MCMs and dinucleotide ZOMs.

### Distance homology searches with large DNA sequences

**Figure 5:**
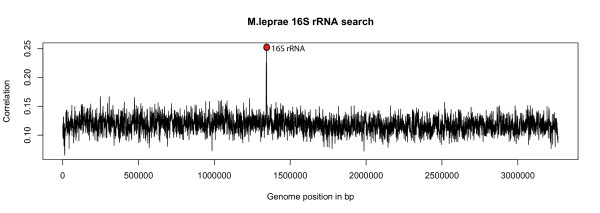
**Homology search/alignment based on heptanucleotide ROFs in *Mycobacterium leprae***. Homology search based on heptanucleotide ROFs in *M. leprae*, using a 1 kbp non-overlapping sliding window compared with a vector consisting of heptanucleotide frequencies taken from 5 kbp of *T. maritima *DNA consisting of 16S, 23S and 5S rRNA genes. The horizontal axis represents nucleotide positions, each point spanning 1 kbp, in the *M. leprae *chromosome, while the vertical axis gives correlation values based on comparisons between the sliding window and the *T. maritima *DNA vector. The marked peak indicates the closest hit, containing corresponding rRNA genes in *M. leprae*. Although *M. leprae *is very distantly related to *T. maritima *(hexanucleotide ZOM score of 0.13) its rRNA genes could be detected using DNA from the corresponding *T. maritima *rRNA genes with the search method based on ROFs.

For the distant homology search the heptanucleotide frequencies of a 5 kbp DNA sequence consisting of rRNA genes taken from *T. maritima *were compared against heptanucleotide frequencies in the *M. leprae *chromosome (hexanucleotide ZOM correlation 0.13 with *T. maritima*) with the sliding window approach described in the Methods section. From Figure 5, a good hit can be observed that achieved a noticeable higher correlation score around position 1.345 mbp than any other place in the genome. BLAST reports the corresponding DNA to be *M. leprae *5S, 16S, 23S rRNA genes with a 100% identity score. The DNA taken from *T. maritima *obtained only a 75% identity score with the *M. leprae *genome, indicating that the ROF method can cope with both large and mutated DNA sequences, while still being fairly fast.

### Plasmid-host similarity analysis

**Figure 6:**
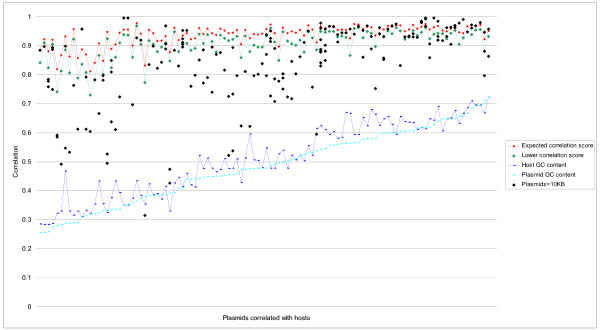
**Plasmid-hosts comparisons based on the tetranucleotide ZOM measure**. Plasmids sized 10 kbp and larger were compared with their corresponding archaeal and bacterial hosts. Plasmid-host correlation values (black dots) were then compared with host average autocorrelation values (expected plasmid-host correlation score, red line) based on 40 kbp sliding windows and tetranucleotide ZOMs. The green line represents lower autocorrelation values, i.e. average autocorrelation values subtracted by standard deviation, while the blue and cyan lines show host and plasmid GC content respectively. The vertical axis represents host bacteria average autocorrelation values (red line), host GC content (blue line), plasmid GC content (cyan), and plasmid-host correlations (black dots). All bacteria and archaea with corresponding plasmids are distributed as points along the horizontal axis and sorted by increasing plasmid GC content from left to right. From the graph it can be observed that GC rich bacteria were more similar to their plasmids in terms of tetranucleotide ZOMs than AT rich bacteria. It can also be noticed that average autocorrelation scores (expected plasmid-host correlation scores) seems to increase and become less volatile for GC rich bacteria than their AT rich counterparts.

Tetranucleotide ZOMs were used to compare plasmids sized 10 kbp and larger with corresponding host genomes, totalling 83 different bacteria and archaea with 108 chromosomes and 179 different plasmids. A minimum plasmid size was used to make the comparisons as accurate as possible between plasmids and hosts using the tetranucleotide ZOM method. Our findings support the claim in [26] that plasmids are, especially AT rich genomes, more distantly related to their host (average correlation of 0.82) than what would be expected from their hosts average autocorrelation values based on correspondingly sized sliding windows (average correlation of 0.94), see Figure 6 (additional file 7 contains a labeled graph) and materials and methods section for details. In addition, we found that plasmid similarity to host, *Y*_*PH*_, correlated well with host average autocorrelation values, *X*_*AAC*_, and GC content, *X*_*GC*_,

*Y*_*PH *_= (-2.75 + 3.34*X*_*AAC *_+ 0.45*X*_*GC*_)^1/3^

*R*^2 ^= *0.4 *(*P *<*0.001*), i.e. higher GC content and chromosomal average ZOM autocorrelation (expected correlation values) values means closer correlation with plasmids. Correlation between *X*_*AAC *_and *X*_*GC *_was *R*^2 ^= *0.4356*. This means that plasmid similarity to host tends to be connected with intra-chromosomal homogeneity and GC content. The bacteria that obtained the highest plasmid-host correlation scores were predominantly soil/free-living, while host-associated and pathogenic typically had the lowest and most varied.

Last, but not least, we calculated plasmid-host similarity based on plasmid size, see materials and methods for details. In short, plasmid-host correlation values were compared to correspondingly sized host average autocorrelation values based on appropriately sized sliding windows. We chose three different sizes for the sliding windows depending on the size of the plasmids. For plasmids sized 10–30 kbp we used a 15 kbp sliding window, 30–70 kbp plasmid comparisons were based on a 40 kbp sliding window, while plasmids sized 70 kbp and above were compared to 100 kbp sliding windows. For the first group, consisting of 32 plasmids sized 15–30 kbp and their respective hosts, we found that the average ZOM autocorrelation value was 0.84. The average of the respective plasmid-host correlations was 0.69. In the next group, consisting of 50 plasmids sized 30–70 kbp, the average ZOM autocorrelation value was 0.93, while the average for the respective plasmid-host correlations was 0.82. For the largest plasmids, sized 70 kbp and above, 97 in total, we found that the average based on ZOM autocorrelation values was 0.97 and the corresponding average plasmid-host correlation was 0.91.

## Discussion

This paper has focused on comparing different statistical methods based on oligonucleotide frequencies. We have paid particular attention to ZOMs by including word sizes from dinucleotides up to hexanucleotides while focusing on tetranucleotides for the ROF and MCM based comparisons. This choice was made because the ZOM method obtained large differences between observed and expected values, or departures [15], which in turn can be regarded as information. The Markov based measures are presumed to give a composite picture of bacterial mechanisms reflected in the chromosome, and in this sense they can be considered to carry a stronger phylogenetic signal than other types of such measures [12-15]. The over- and under-expression of certain oligonucleotides can be caused by DNA structural conformations, DNA base stacking energies, codon preference, and protein binding sites including transcription/restriction sites, DNA replication and repair, etc. [12-17].

While MCMs are thought to be affected by similar pressures, they are likely to be much less so compared with ZOMs, since they approximate tetranucleotide frequencies based on observed transition probabilities from di- and trinucleotide distributions. In other words, tetranucleotide MCMs assume that tetranucleotide frequencies will have the same transition probabilities from trinucleotide frequencies as trinucleotide frequencies have from dinucleotide frequencies. Since large parts of bacterial DNA codes for proteins the tetranucleotide MCM transition probabilities will, to a large extent, be affected by codon distributions. Differences between observed tetranucleotide frequencis and expected, based on MCMs, will therefore reflect additional influences than codon preference. Phylogenetic comparisons based on tetranucleotide ROFs are more directly affected by GC-content and codon usage than the other measures since they are only normalized by sequence size. In other words, there is no assumption of discrepancy from any expected statistical distribution. Comparisons between archaea and bacteria based only on oligonucleotide frequencies are thus measuring differences in relative oligonucleotide occurences. Archaea and bacteria with similar genes and GC content might therefore be considered more alike regardless of phylogenetic relationship [2,3].

The first part of this analysis was motivated by the observation that not much has been stated about possible errors for the different statistical measures mention above. A fake genome was created with a uniform distribution of nucleotides comparable to *E. coli *in both size and GC content. Although most of the measures showed low correlation between the fake genome and the 581 real genomic DNA sequences, dinucleotide ZOMs tended to give some very high correlation values. For instance, *M. hungatei *strain JF-1 achieved a correlation value of 0.81 with the completely random genome. This is the same value obtained for *Mycoplasma pneumoniae *strain M129 (0.82 mbp, 40% GC) compared with *Mycoplasma genitalium *strain G37 (0.58 mbp, 31.7% GC). For trinucleotide to hexanucleotide ZOMs the corresponding correlation values with the random genome were 0.3, 0.11, 0.05 and 0.03 respectively. We believe that the high correlation values that dinucleotide ZOMs obtained are due to the small oligonucleotide size considered in the method. Using dinucleotides there are only *4*^2 ^= *16 *possible combinations which means that a smaller amount of DNA is needed to get an understanding of how the dinucleotide distributions progresses. For hexanucleotides there are *4*^6 ^= *4096 *possible combinations resulting in large vectors containing far more detail, but also the requirement of substantially larger amounts of DNA. Our conclusions are therefore that dinucleotide ZOMs can be used to give some limited inference of phylogenetic similarity, with an increased risk of false positives, between small DNA sequences (250–500 bp), whilst penta- and hexanucleotide ZOMs and the MCM measure are best-suited for whole genome comparisons due to the requirements of large amounts of DNA. We find that tetranucleotide ZOMs are good all-round measures that can be used to measure similarity between both relatively small DNA sequences, 3 to 4 kbp in size, and larger, while still giving reliable comparison results and information.

Comparing the statistical measures against real genomes, we found that the ZOM measures became increasingly ordered with larger oligonucleotide sizes, with the MCM measure performing comparable to the penta- and hexanucleotide ZOM measures for the genomes tested. The MCM and ROF measures produced the most reliable rankings of *S. aureus *and *B. subtilis*, with the MCM slightly better than the ROF measure. Results obtained with the ROF and dinucleotide ZOM measures were, in general, varied, and it is our impression that the MCM, and penta- and hexanucleotide ZOMs were the most robust measures in terms of phylogeny. The MCM measure seemed to have a better ability to detect farther relatives than the other measures, although more genomes must be tested to confirm this claim.

Presumed extensive DNA exchange with species from all kingdoms of life [20,21] has made phylogenetic characterization of the hyperthermophilic *T. maritima *difficult, which may explain the different results obtained with the statistical measures tested.

Autocorrelation profiles are often used in whole genome analysis to search for regions of special interest. These regions are thought to be composed of foreign or conserved DNA. We used *B. subtilis *and *T. maritima *in our examples since they are presumed to have undergone extensive horizontal transfer [20-23]. The different ZOM autocorrelation profiles are similar though differences can be detected. For instance, dinucleotide ZOMs contains far less detail than hexanucleotide ZOMs which, on the other hand, have low average autocorrelation values indicating that the sliding windows used (sized 5 kbp) are too small for that particular measure. The lack of detail for dinucleotide ZOMs may be due to the opposite reason, namely that the sliding window is too large. As mentioned above, dinucleotides come in 16 different combinations and, assuming a uniform distribution, each dinucleotide will be repeated for every 16 base pairs. Hexanucleotides have 4096 different combinations and each hexamer will therefore be expected to appear for every 4096 base pairs, again assuming a uniform distribution. This implies that the size of the oligonucleotide used for profiles should depend on the sliding window. Another possibility for the difference in detail with respect to autocorrelation profiles is that smaller oligonucleotides lack information present in larger ones.

Turning now to tetranucleotide ROFs and MCM based autocorrelation profiles, illustrated in Figure 3, we find that the profiles based on tetranucleotide ROFs appear to be quite similar to ZOMs. In these particular examples ZOMs seem to carry more detail even though tetranucleotides are used in both profile types. Similarly to the hexanucleotide ZOM profiles, the tetranucleotide MCM autocorrelation profiles have also low average autocorrelation scores. As explained previously, this is thought to be caused by low departure or discrepancy values between expected and observed frequencies. In other words, this could indicate that tetranucleotide MCMs need larger sliding windows. Although most of the other dips were missing from the *T. maritima *profile some unique ones were also found, most notably at positions 320 kbp and 1.350 mbp. Considering the fact that the dip located at position 320 kbp appeared to be linked with the ABC transporter system, as shown above, we believe that tetranucleotide MCMs presents complimentary information to the other measures used. Increasing the sliding window size from 5 kbp to 20 kbp for both the hexanucleotide ZOMs and tetranucleotide MCM based profiles, increased average autocorrelation scores, but reduced detail in the respective profiles noticeably, even though the sliding windows were overlapping every 5 kbp (see Figure 4). Thus, the tetranucleotide ZOM measure with 5 kbp sliding windows, overlapping every 2.5 kbp, seems to be the best measures in terms of sliding window size and detail.

ROFs stand out from the other measures when it comes to homology searches. Large DNA sequences, many kbp or mbp in size, can be used and since the methods are not seed-based homologous DNA sequences with many mutations may still be detected. This was demonstrated by taking the DNA sequence in *T. maritima *containing a set of rRNA genes (see results section and Figure 5). Since the sequence is close to 5 kbp we chose a 1 kbp sliding window and heptanucleotide frequencies. In terms of homology searches based on ROFs, fixed sized words larger than tetranucleotides have to be used in order to get sufficient precision. Actually, this process is exactly the same as the one used for the *T. maritima *autocorrelation profile (see Figure 3 and results section) with the exceptions that the global vector now contains the DNA word frequencies from the rRNA genes taken from *T. maritima*, the sliding window is only 1 kbp long, and heptanucleotides are used instead of tetranucleotides. The frequency-based search program used was only 150 lines of C++ code, and performed the search in less than 7 seconds on a 3 gHz Pentium 4 computer. Although the method is relatively fast, easy to implement, and can handle sequences from 50–60 bp up to millions of bp, it suffers from the fact that the hit positions are never more accurate than the sliding window. Making the method more accurate means that the sliding windows must overlap more often, thereby removing the speed advantage gained in comparing large DNA sequences. This can be remedied by combining the ROF method with a secondary method, like a seed based search algorithm.

While ROFs may be the most appropriate measure for homology searches, ZOMs and the MCM measure may carry stronger phylogenetic signals due to reasons explained above. Tetranucleotide ZOMs were shown above to give reasonably good results in terms of size, reliability and detail. We therefore used that measure to compare plasmids with their hosts. As has been previously noted [26], we find that plasmids are much less correlated with their hosts than what would be expected from autocorrelation scores based on similarly sized sliding windows (see results section). Large plasmids tend to be closer to their hosts, at least in terms of tetranucleotide ZOMs, than smaller plasmids. Whether this is due to the increased amount of DNA available for the comparisons or that larger plasmids are, for some reason, more affected by host amelioration processes is not known. The fact that GC rich bacteria and archaea with high tetranucleotide ZOM average autocorrelation values (plasmid-host similarity expectations) attain better correlation scores with their plasmids may be caused by better DNA maintenance in such bacteria and/or lower mutation rates [27]. Since many host-associated AT rich bacteria tend to lose their DNA replication and repair systems [27] we believe that this may be one explanation for the lower plasmid-host correlation scores.

## Conclusion

In this paper, we have investigated different statistical methods based on oligonucleotide frequencies, to compare microbial DNA from various genomes. Dinucleotide ZOMs can, with care, be used for small DNA sequences, while hexanucleotide ZOMs and MCMs seems more reliable for large DNA sequences and whole genome comparisons.

ZOM, MCM and ROFs were found to be complimentary measures in terms of autocorrelation profiles. The tetranucleotides ZOM measure was found to be the most versatile in terms of detail, error, and sliding window size when used for DNA profiling.

The ROF measure was the best of the measures for fast distant homology searches with large DNA sequences.

Finally, based on the findings from the autocorrelation profiles, tetranucleotide ZOMs were used to look at similarities between a selection of plasmids, sized 10 kbp and larger, and their corresponding hosts. It was found that plasmid-host DNA similarity correlated with intra-chromosomal homogeneity of the host, and GC content, i.e. the more GC rich and homogeneous a genome was, the more similar were the corresponding plasmids. Plasmids were found to be more dissimilar to their hosts than expected, and similarity seemed also to be related to plasmid size.

## Methods

### Sequence data

Genomic DNA sequences consisting of 581 chromosomes and plasmids were downloaded from GenBank [28], September 2006. An artificial and completely random chromosome was created with similar size (5.3 mbp) and GC content (50%, uniformly distributed) as typical for an *E. coli *genome. The random DNA sequence was compared to all 581 sequences above to examine the reliability of the different measures.

*B. subtilis *and *T. maritima *were used as examples in the DNA profiles due to their presumed history of considerable DNA acquisition [20-23].

### Formulas and calculations

Let *F(.) *denote a frequency function, where *F*_*x*_*(A)*, *F*_*x*_*(G)*, *F*_*x*_*(C)*, or *F*_*x*_*(T) *gives the relative frequency of the nucleotides A, G, C and T with respect to the DNA sequence *x*. GC-skew can now be expressed as

$$\frac{F_{x}(G)-F_{x}(C)}{F_{x}(G)+F_{x}(C)}$$

We also allow *F(.) *to give relative frequencies for overlapping oligonucleotides. That is, *F*_*x*_*(w*_1_*w*_2_...*w*_*n*_*) *gives the relative frequency of the DNA word *w*_1_*w*_2_...*w*_*n *_in sequence *x*. The formula used for odds-ratio is

$$\frac{O(w_{1}w_{2}...w_{n})}{E(w_{1}w_{2}...w_{n})}$$

Where *O(w*_1_*w*_2_...*w*_*n*_*) *represents observed *n*-word frequencies, and *E(w*_1_*w*_2_...*w*_*n*_*) *expected, according to a given statistical distribution, i.e. ZOM or MCM.

With this notation the odds-ratio formula for ZOMs can be written as:

$$\frac{F_{x}(w_{1}\ldots w_{n})}{F_{x}(w_{1})F_{x}(w_{2})\cdots F_{x}(w_{n})}$$

That is, the relative frequency of the word *w*_1_...*w*_*n *_divided by the relative frequency of each of its individual nucleotides *w*_1_,*w*_2_,...,*w*_*n *_with respect to DNA sequence *x*.

MCMs can be calculated analogously with the following formula:

$$E(w_{1}\ldots w_{n})=\frac{F_{x}(w_{1}\ldots w_{n-1})F_{x}(w_{2}\ldots w_{n})}{F_{x}(w_{2}\ldots w_{n-1})}$$

The formula states that the transition probability for *n *mers from *n-1 *mers is the same as for *n-1 *mers from *n-2 *mers.

Formula (2) gives the following odds-ratio formula for MCM's

$$\frac{F_{x}(w_{1}\ldots w_{n})F_{x}(w_{2}\ldots w_{n-1})}{F_{x}(w_{1}\ldots w_{n-1})F_{x}(w_{2}\ldots w_{n})}$$

Comparisons between two DNA sequences with respect to oligonucleotide frequencies were carried out as follows: each sequence was associated with a vector containing *4*^*n *^entries and thus corresponding to the relative frequency of every possible combination of an oligonucleotide consisting of *n *nucleotides. The two resulting vectors are then compared with the following correlation formula:

$$\frac{\sum_{i}\left(x_{i}-\bar{x})(y_{i}-\bar{y}\right)}{\sqrt{\sum_{j}{\left(x_{j}-\bar{x}\right)}^{2}\sum_{k}{\left(y_{k}-\bar{y}\right)}^{2}}}$$

Where

*x*_*i *_= *F*_*x *_^*i*^*(w*_1 _^*i*^...*w*_*n *_^*i*^*)*

*y*_*i *_= *F*_*y *_^*i*^*(w*_1 _^*i*^...*w*_*n *_^*i*^*)*

denotes the frequency of *n*-word *i *for DNA sequences *x *and *y*, with respective averages given by

$$\bar{x}=\frac{1}{N}\sum_{i}^{N}x_{i}$$

$$\bar{y}=\frac{1}{N}\sum_{i}^{N}y_{i}$$

*N *= *4*^*n *^denotes the number of all possible combinations of a DNA word with length *n*.

The comparisons based on ZOMs and MCMs are carried out in the same manner, but with the right hand side of equations (4) and (5) substituted with formulas (1) and (2), respectively. Formula (3), or the Pearson linear correlation formula, can be thought of as a measure describing the angle between vectors *x *and *y*. Values approaching 1 (or -1) means that the vectors are linear and parallel (or anti-parallel), while values close to 0 means that they are perpendicular and thus completely different. This approach was chosen because of its emphasis on the direction of the vectors, making the measure scale invariant. Sequence comparisons based on formula (3) will hereafter be referred to as correlation scores/values, or simply scores.

### Autocorrelation profiles and distant homology search

The DNA profiles were made by first obtaining a global vector consisting, in turn, of tetranucleotide ROF, MCM, and (di-, tetra-, and hexanucleotide) ZOM values for the complete *B. subtilis *and *T. maritima *chromosomes. Formula (3) was then used to compute correlation scores between the global vector and a local vector consisting of values based on a 5/20 kbp sliding window, overlapping every 2.5/5 kbp, within the same chromosome for each measure. The correlation values obtained from such comparisons are referred to as autocorrelation scores/values. Low autocorrelation values are hypothesized to be caused by conserved, foreign, or horizontally transferred regions [14,16,17]. Such regions were singled out and BLASTed against the NCBI database *nr*.

The distant homology search was performed similarly to autocorrelation profiles, but with the global vector now containing the heptanucleotide frequencies found in the *T. maritima *rRNA genes. The global vector was then compared to local vectors consisting of heptanucleotide frequencies taken from a 1 kbp non-overlapping sliding window throughout the *M. leprae *chromosome.

### Plasmids-host correlations

Comparisons between plasmids and host chromosomes were performed with similar methods as those used for the DNA profiles above, but with tetranucleotide ZOMs. A non-overlapping 40 kbp sliding window was chosen, to correspond approximately to average plasmid sizes. Average autocorrelation scores were found by computing the correlation scores between a global vector and local vectors representing each sliding window. These correlation scores were progressively added together and divided by the total number of sliding windows to obtain the average autocorrelation value. Plasmid-host correlation and chromosome average autocorrelation values were based on smallest plasmids and 40 kbp sliding windows respectively. Regression analysis was performed on the correlation scores between the smallest plasmids and their respective chromosomes, host average autocorrelation values and host GC-content. The following formula was used:

*Y*_*PH *_= (*α *+ *β*_0_*X*_*AAC *_+ *β*_1_*X*_*GC*_)^*λ*^

*Y*_*PH *_describes plasmid-host correlation, *λ *is the transform coefficient, *α *is the intercept coefficient, *β*_0 _is the coefficient for host average autocorrelation scores (*X*_*AAC*_) and *β*_1 _is the host GC (*X*_*GC*_) content coefficient.

The size oriented plasmid-host similarity analysis was carried out as follows: Plasmid-host correlation scores, plasmids sized in three categories: 10–30 kbp, 30–70 kbp and 70 kbp and larger, were compared with host average autocorrelation values (expected correlation scores), based on the sliding window approach described above, but with sliding windows sized 15, 40 and 100 kbp according to plasmid sizes. The difference was then computed for each of the three groups according to the following formula:

$$m_{x}=\frac{1}{N}c_{x}$$

*m*_*x *_is the average value calculated for either *x *= *a *or *x *= *p*, where *a *= average autocorrelation values and *p *= plasmid-host correlation values, *N *is the number of comparisons, while *c*_*x *_represents comparison values based on *x*, with *x *= *a *or *x *= *p *as above.

All methods described above were implemented in different computer programs and run on a standard desktop PC. Visualisation of the results obtained from the computer programs and statistical analysis was carried out with the program R [29]. The source code for the computer programs can be obtained from the corresponding author at request.

## Authors' contributions

JB planned the project, made the computer programs and drafted the manuscript. ES contributed to the statistical analysis and interpretation of data. DU carried out analysis, helped draft, revise, and polish the manuscript and coordinated the project. All authors have read and approved the final manuscript.

## Supplementary Material

Additional file 1 **Random genome compared with sequenced bacterial and archaeal genomes using ZOMs**. A detailed Excel table and plot of Figure 1 containing labels of all chromosomes and plasmids correlated (column A) with a random DNA sequence using di- to hexanucleotide ZOM (columns E-I, respectively), MCM and ROF measures (columns J and K, respectively). DNA sequence size and AT content is respectively found in columns B and C. Click here for file

Additional file 2 ***B.subtilis *compared with sequenced bacterial and archaeal genomes using ZOMs**. A detailed plot containing labels of all chromosomes and plasmids (column A) correlated with *B. subtilis *using di- to hexanucleotide ZOM (columns E-I, respectively), MCM and ROF measures (columns J and K, respectively). DNA sequence size and AT content is respectively found in columns B and C. Click here for file

Additional file 3 ***Y. pestis *compared with sequenced bacterial and archaeal genomes using**. A detailed Excel table and plot containing labels of all chromosomes and plasmids (column A) correlated with *Y. pestis *using di- to hexanucleotide ZOM (columns E-I, respectively), MCM and ROF measures (columns J and K, respectively). DNA sequence size and AT content is respectively found in columns B and C. Click here for file

Additional file 4 ***P.aureginosa *compared with sequenced bacterial and archaeal genomes using ZOMs**. A detailed Excel table and plot containing labels of all chromosomes and plasmids (column A) correlated with *P. aeruginosa *using di- to hexanucleotide ZOM (columns E-I, respectively), MCM and ROF measures (columns J and K, respectively). DNA sequence size and AT content is respectively found in columns B and C. Click here for file

Additional file 5 ***S.aureus *compared with sequenced bacterial and archaeal genomes using ZOMs**. A detailed Excel table and plot containing labels of all chromosomes and plasmids (column A) correlated with *S. aureus *using di- to hexanucleotide ZOM (columns E-I, respectively), MCM and ROF measures (columns J and K, respectively). DNA sequence size and AT content is respectively found in columns B and C. Click here for file

Additional file 6 ***T.maritima *compared with sequenced bacterial and archaeal genomes using ZOMs**. A detailed Excel table and plot containing labels of all chromosomes and plasmids (column A) correlated with *T. maritima *using di- to hexanucleotide ZOM (columns E-I, respectively), MCM and ROF measures (columns J and K, respectively). DNA sequence size and AT content is respectively found in columns B and C. Click here for file

Additional file 7 **Plasmid-host comparisons using tetranucleotide ZOMs**. A more detailed plot of Figure 6 containing the names of all bacterial and archaeal genomes compared with corresponding plasmids sized 10 kbp and larger. Click here for file
