# Supplementary figures and images for: Reliability and applications of statistical methods based on oligonucleotide frequencies in bacterial and archaeal genomes
Source: BMC Genomics. 2008 Feb 28;9:104. doi: 10.1186/1471-2164-9-104 (PMC2289816; doi:10.1186/1471-2164-9-104)

### Plasmid-host comparisons

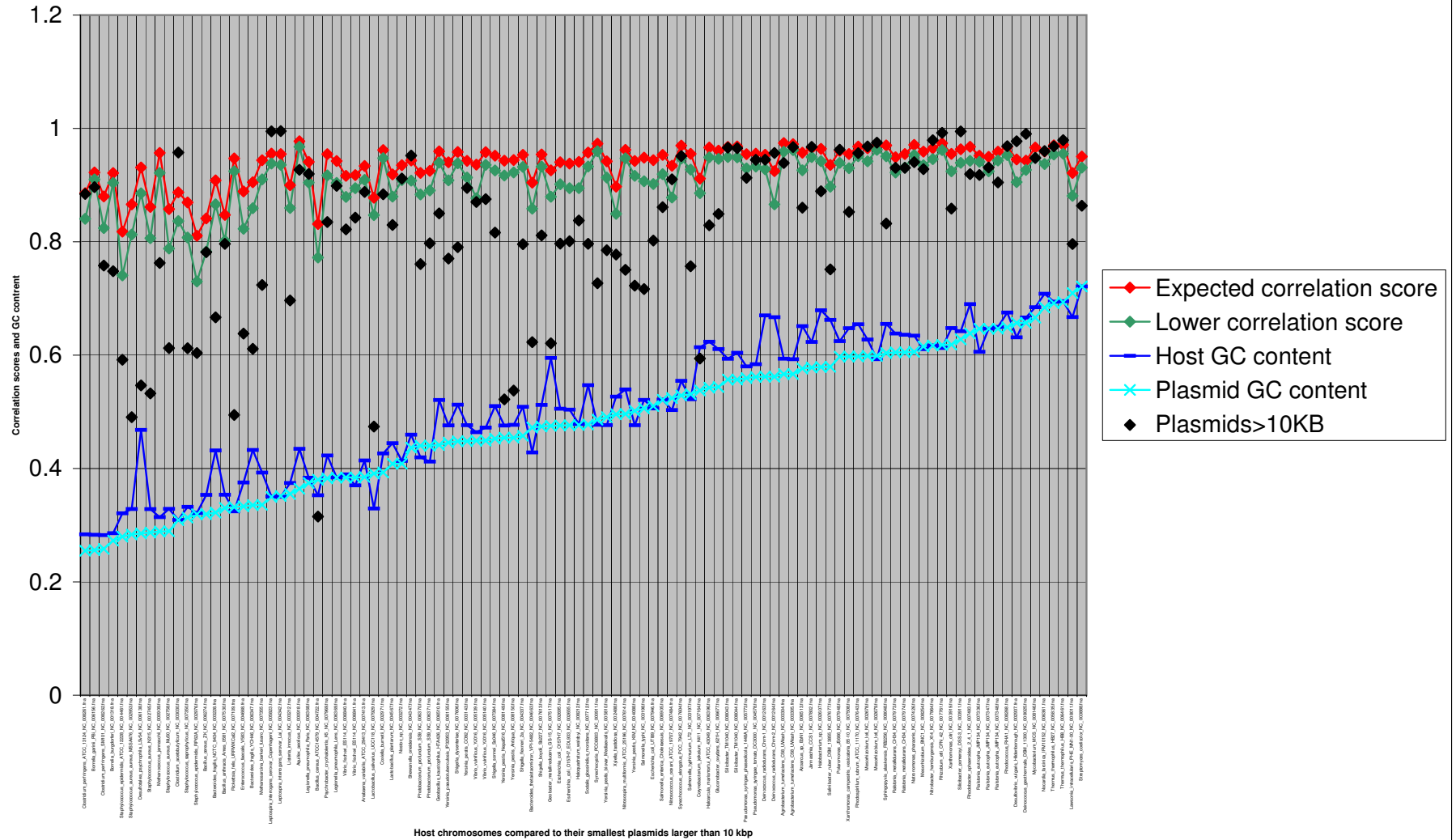

Supplement: Additional file 7 — Plasmid-host comparisons using tetranucleotide ZOMs. A more detailed plot of Figure 6 containing the names of all bacterial and archaeal genomes compared with corresponding plasmids sized 10 kbp and larger. [file 1471-2164-9-104-S7.pdf]
